# Intraoperative management of suspected ocular venous air embolism (OVAE) during vitrectomy for retinal detachment

**DOI:** 10.1016/j.ajoc.2025.102485

**Published:** 2025-12-05

**Authors:** Alireza Helal Birjandi, Ermioni Panidou-Marschelke, Lisa-Marie Horn, Kilian Arlt, Carsten Framme, Jan Tode

**Affiliations:** a Universitätsklinik für Augenheilkunde, Medizinische Hochschule Hannover (MHH), Germany; b Klinik für Anästhesiologie und Intensivmedizin, Medizinische Hochschule Hannover (MHH), Germany

## Abstract

**Objective:**

report a rare case of ocular venous air embolism (OVAE) occurring during pars plana vitrectomy for retinal detachment and to highlight key intraoperative management steps that led to a successful outcome.

**Observations:**

A 57-year-old patient undergoing 23-gauge vitrectomy developed abrupt hemodynamic instability during air-fluid exchange. Intraoperative signs—including sudden drops in end-tidal CO_2_ and oxygen saturation—prompted suspicion of air embolism, likely due to suprachoroidal air infusion via a dislodged trocar. Immediate cessation of air infusion, internal tamponade, cardiovascular support, and surgical revision of the infusion system stabilized the patient. Postoperative imaging supported right heart strain consistent with venous air entry. The retinal detachment was successfully treated, and the patient recovered without long-term systemic or ocular complications.

**Conclusion:**

This case underscores the potentially fatal risk of OVAE during vitrectomy and the importance of early recognition, prompt intervention, and secure cannula placement. Increased awareness and surgical vigilance are critical for prevention and management of this underreported complication.

## 1 Case presentation

A 57-year-old male presented with a macula-off rhegmatogenous retinal detachment from 8:00 to 3:30 and, with a horseshoe tear at 2:00, two weeks of progressive symptoms, acute central vision loss since the previous day, and an incipient cataract. Best-corrected visual acuity was hand motion in the right eye. Intraocular pressure (IOP) was normal. Combined phacoemulsification, intraocular lens implantation, and 23G vitrectomy under general anesthesia were scheduled.

**Fig. 1:**
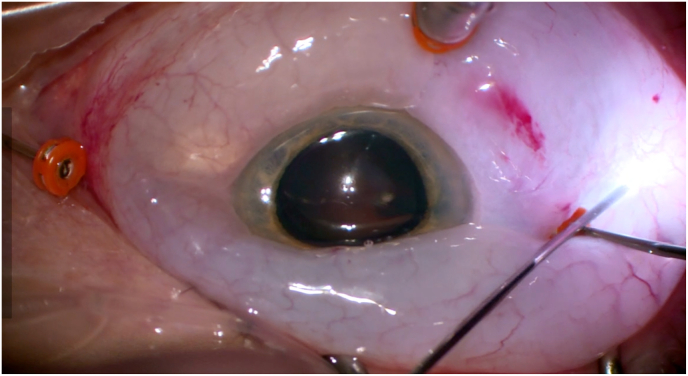
Loosening of trocar cannulas with massive conjunctival edema.

**Fig. 2:**
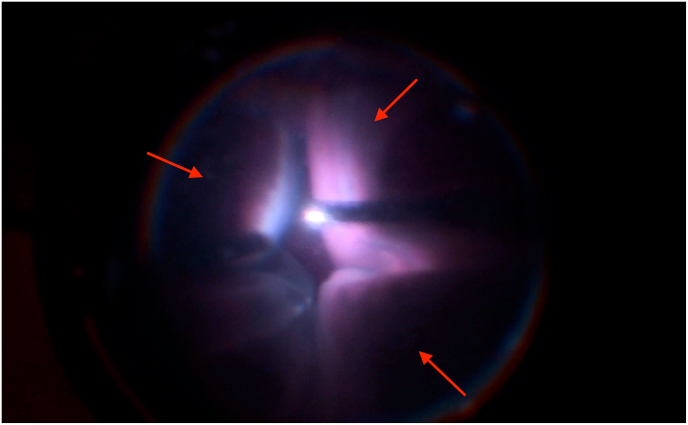
Sudden development of a 360-degree choroidal effusion.

Vitrectomy was performed via pars plana using three transconjunctival, unsutured trocar cannulas. During peripheral vitrectomy, subconjunctival fluid was observed, indicating initial cannula displacement. We assume that this fluid tracked through the unsutured sclerotomies, creating a positive-feedback loop that led to massive conjunctival edema. The resulting edema progressively loosened all three trocars, including the infusion line (Fig. 1). To evacuate the accumulated fluid, a temporal conjunctival incision was made. After trocar reimplantation, vitrectomy was completed into the far periphery. Shortly thereafter, a dome-shaped 360-degree non-hemorrhagic choroidal effusion developed (Fig. 2), with anterior chamber shallowing.

To stabilize the posterior segment, an air-fluid exchange was initiated. Intraoperative assessment revealed secondary displacement of the correctly reimplanted infusion cannula. Immediately, mean arterial pressure dropped to 58 mmHg, end-tidal CO_2_ fell from 47 to 14 mmHg, SpO_2_ decreased from 97 % to 73 %, and heart rate rose from 66 to 101 bpm, indicating systemic air embolism likely caused by suprachoroidal air entry via the displaced cannula.

Air infusion was stopped, viscoelastic injected, FiO_2_ increased to 100 %, and vasopressors administered. An attempted intraoperative Echocardiography was inconclusive due to obesity and surgical position.

**Fig. 3:**
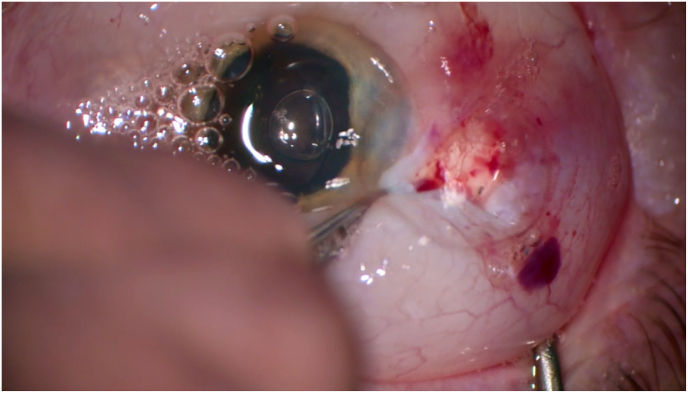
Drainage of air and fluid through sclerotomies, without signs of hemorrhagic complication.

**Fig. 4:**
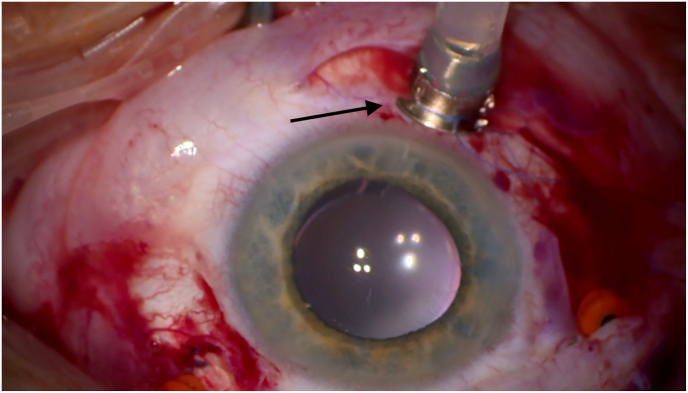
Replacement of the infusion port with a sutured longer cannula.

Intraoperative inspection revealed infusion cannula displacement with presumed suprachoroidal air infusion. To decompress intraocular pressure, two posterior sclerotomies were made, with air and fluid egressing without hemorrhage (Fig. 3). An anterior chamber maintainer was placed, and the infusion port replaced with a longer, sutured cannula (Fig. 4). Hemodynamic parameters stabilized within 10 min.

Cryopexy was applied to the tear, followed by perfluorocarbon instillation, subretinal fluid drainage, and 5000 cSt silicone oil tamponade. Final inspection showed mild nasal and temporal choroidal elevation without evidence of progression during the final surgical period.

Postoperatively, the patient was monitored in the ICU. Echocardiography showed an acute right heart strain with right ventricular dilation and septal flattening (D-sign). CT angiography ruled out pulmonary embolism but revealed pulmonary edema. Cardiac enzymes (CK and troponin-T) showed mild elevation.

The patient was discharged on postoperative day 2 in stable condition. Visual acuity was 20/160, IOP 14 mmHg. Retina remained attached under silicone oil with some choroidal folds but no effusion.

## 2 Discussion

Air embolism during vitrectomy, recently termed Presumed Air by Vitrectomy Embolisation (PAVE), is rare but potentially fatal. It may occur when pressurized air enters the suprachoroidal space and escapes into systemic circulation via torn vortex veins with displaced small-gauge cannulas.^1^ Despite its severity, PAVE remains underrecognized among vitreoretinal surgeons, with one survey finding only 20 % of vitreoretinal specialists were aware of OVAE. This lack of awareness contributes to its high mortality rate; 9 of 13 reviewed cases were fatal.^2^ Given its lethality, prevention is critical. the surgical team must perform a safety check to confirm the proper placement of the infusion cannula before starting a fluid-gas exchange. If a choroidal detachment occurs, air infusion must be stopped immediately. In high-risk cases, such as trauma repair, using a secured infusion cannula and a precordial Doppler monitor is beneficial.^3^ If VAE is suspected, the infusion should be stopped, 100 % oxygen applied, and the patient placed in the Trendelenburg position.^4^ Cardiopulmonary resuscitation may be necessary, and transfer to a hospital with ECMO capabilities can be lifesaving.^3^

In our case, secondary misalignment of the infusion cannula likely led to retrograde suprachoroidal air infusion and systemic air embolism, evidenced by sudden hypocapnia, hypoxemia, and tachycardia. Prompt recognition and intervention stabilized the patient.

## 3 Conclusion

This case highlights the need for early recognition and management of OVAE during vitrectomy. Vital considerations include proper cannula placement, hemodynamic monitoring during air infusion, and close coordination with anesthesiology.

## CRediT authorship contribution statement

**Alireza Helal Birjandi:** Writing – review & editing, Writing – original draft, Resources, Data curation, Conceptualization. **Ermioni Panidou-Marschelke:** Validation, Supervision, Project administration, Conceptualization. **Lisa-Marie Horn:** Writing – review & editing. **Kilian Arlt:** Writing – review & editing, Supervision. **Carsten Framme:** Validation, Supervision. **Jan Tode:** Validation, Supervision, Project administration, Conceptualization.

## Consent

Written informed consent obtained.

## Funding

No financial support.

## Declaration of competing interest

The authors declare that they have no known competing financial interests or personal relationships that could have appeared to influence the work reported in this paper.
